# Correlation Between Resting Testosterone/Cortisol Ratio and Sound-Induced Vasoconstriction at Fingertip in Men

**DOI:** 10.3389/fphys.2018.00164

**Published:** 2018-03-06

**Authors:** Yuuki Ooishi

**Affiliations:** NTT Communication Science Laboratories, NTT Corporation, Atsugi, Japan

**Keywords:** sound, testosterone, cortisol, sympathetic nervous system, blood volume pulse response, vasoconstriction

## Abstract

A sound-induced sympathetic tone has been used as an index for orienting responses to auditory stimuli. The resting testosterone/cortisol ratio is a biomarker of social aggression that drives an approaching behavior in response to environmental stimuli, and a higher testosterone level and a lower cortisol level can facilitate the sympathetic response to environmental stimuli. Therefore, it is possible that the testosterone/cortisol ratio is correlated with the sound-induced sympathetic tone. The current study investigated the relationship between the resting testosterone/cortisol ratio and vasoconstriction induced by listening to sound stimuli. Twenty healthy males aged 29.0 ± 0.53 years (mean ± S.E.M) participated in the study. They came to the laboratory for 3 days and listened to one of three types of sound stimuli for 1 min on each day. Saliva samples were collected for an analysis of salivary testosterone and cortisol levels on the day of each experiment. After the collecting the saliva sample, we measured the blood volume pulse (BVP) amplitude at a fingertip. Since vasoconstriction is mediated by the activation of the sympathetic nerves, the strength of the reduction in BVP amplitude at a fingertip was called the BVP response (finger BVPR). No difference was observed between the sound-induced finger BVPR for the three types of sound stimuli (*p* = 0.779). The correlation coefficient between the sound-induced finger BVPR and the salivary testosterone/cortisol ratio within participants was significantly different from no correlation (*p* = 0.011) and there was a trend toward a significance in the correlation between the sound-induced finger BVPR and the salivary testosterone/cortisol ratio between participants (*r* = 0.39, *p* = 0.088). These results suggest that the testosterone/cortisol ratio affects the difference in the sound-evoked sympathetic response.

## Introduction

Sound-induced sympathetic responses have been widely examined in psychophysiological studies (Minami et al., 1993; Hay et al., 1997; Péréon et al., 2001; Ooishi and Kashino, 2012; Sato and Ooishi, 2012). The strength of sympathetic nerve activity has commonly been evaluated by measuring electrocardiograms (Watanabe et al., 2015), electrodermal activity (Turpin et al., 1999), and photoplethysmographs (PPGs) (Ooishi and Kashino, 2012), from which the heart rate, skin conductance response, and blood volume pulse (BVP) are calculated. By using these measurements, the degree of sound-induced emotion (Gomez and Danuser, 2004; Salimpoor et al., 2009), and that of habituation to sound stimuli (Vila et al., 2007) have been investigated. In particular, the reduction in BVP amplitude induced by sound stimuli has been used as an index for orienting responses to auditory stimuli (Ooishi and Kashino, 2012; Sato and Ooishi, 2012; Lin et al., 2014). Therefore, sound-induced sympathetic responses have been considered a powerful tool with which to investigate the effect of auditory stimulation on human beings.

Cortisol, which is known as a glucocorticoid in humans, has been well-described as a stress-related steroid hormone. Previous studies have demonstrated that stress tasks increase both the heart rate (HR) and the cortisol level in plasma or saliva (Kudielka et al., 2004; Rimmele et al., 2007). However, as regards the interaction between cortisol and sympathetic activity, the application of glucocorticoid agonist reduces the sympathetic outflow in human beings (Golczynska et al., 1995) and in rats (Brown and Fisher, 1986). These suggest that glucocorticoid works as a suppressor for the sympathetic nerves even though its level is enhanced with stress similar to the HR. In contrast, testosterone has a positive effect on the sympathetic nerves. Testosterone treatment enhances the noradrenaline pressor response in cats (Bhargava et al., 1967). The administration of testosterone increases the muscle sympathetic nerve activity in human beings (Miner et al., 2013) and recovers from the castration-induced decrease in the noradrenergic innervation of rat vas deferens (Lara et al., 1985). From these previous studies, it could be suggested that a higher level of testosterone with a lower level of cortisol facilitates the sympathetic response.

The amygdala can be considered an important region for sound stimuli to induce the sympathetic tone. An earlier study using rats demonstrated that auditory stimulation induces an increase in the firing rate of neurons in the lateral amygdala (LA) as well as in the medial geniculate body (MGB) (Bordi and LeDoux, 1992), and the auditory cortex (AC) has neuronal projections to the LA (Kraus and Canlon, 2012). On the other hand, the central nucleus of the amygdala (CeA) directly (Saha et al., 2005) or indirectly (Saha, 2005) activates the rostral ventrolateral medulla (RVLM). Because the RVLM is the primary regulator of the sympathetic nervous system that controls noradrenergic cardiovascular activity (Kumagai et al., 2012), it could be suggested that the neural connection between the auditory-related regions (MGB and AC) and the amygdala causes the activation of the RVLM neurons that induce the cardiovascular sympathetic tone, when auditory stimulation occurs.

It has been widely demonstrated that the amygdala is susceptible to cortisol and testosterone. Earlier studies using rats have revealed that the amygdala has an ample body of androgen (Sar et al., 1990) and glucocorticoid (Johnson et al., 2005) receptors. Testosterone and cortisol in human beings have also been shown to be associated with amygdala activity. It is already well-known that the fight-or-flight reaction is instigated by the sympathetic nervous system (Johnson et al., 1992), while approaching (fight) or avoidant (flight) behavior is facilitated in the amygdala by testosterone and cortisol, respectively (Terburg et al., 2009). Therefore, the testosterone/cortisol ratio can be regarded as a biomarker for social aggression that drives approaching behavior in response to environmental stimuli (Terburg et al., 2009). In addition, the activation of the amygdala by angry faces vs. happy faces is positively correlated with the resting testosterone/cortisol ratio (Hermans et al., 2008). Taken together, this suggests that the testosterone/cortisol ratio is involved in the sympathetic nervous system-amygdala relationship that regulates the orienting response to environmental stimuli.

Thus, it can be hypothesized that the sound-induced cardiovascular sympathetic response is correlated with the basal testosterone/cortisol ratio. Since the fingertip vasoconstriction represented as a decrease in the BVP amplitude is regarded as a purely noradrenergic cardiovascular sympathetic tone (Grote et al., 2003), this study examined the relationship between the resting testosterone/cortisol ratio and the degree of the decrease in the BVP amplitude induced by sound stimuli. The decrease in the finger BVP amplitude was defined as the finger BVP response (finger BVPR).

## Materials and methods

### Ethics statement

All methods and procedures in this study were approved by the Ethics and Safety Committees of NTT Communication Science Laboratories, and were in accordance with the Declaration of Helsinki.

### Participants

All procedures were conducted in accordance with the Declaration of Helsinki and approved by the Ethics Committee of NTT Communication Science Laboratories. Twenty healthy males aged 29.0 ± 0.53 years (mean ± S.E.M) participated in this study. All of the participants had normal hearing ability. The participants were informed that they would hear several sounds including an aversive one. They gave written consent, which was approved by the Ethics Committee of NTT Communication Science Laboratories. They were paid for their participation. The experiments were performed in a sound-insulated listening room. Participants sat on a sofa and were encouraged to relax. The experiments were conducted between 15:00 and 17:00 h to minimize the effect of circadian rhythms.

### Sound stimuli

In this study, three types of sounds were used as experimental sound stimuli, the details of which are described in a previous study (Ooishi and Kashino, 2012). Briefly, the sound of scratching a blackboard 20 times was recorded binaurally with a dummy head that had recording microphones at the location of the eardrums. The sampling rate was 96 kHz and 24-bit quantization was used. This sound was named type 1. Other type 2 or 3 sounds were produced by MATLAB (The MathWorks, USA). A fast Fourier transformation (FFT) was performed for each type 1 scratching sound. After randomizing the phase domain, the inverse FFT was computed and the amplitude envelope was calibrated so that it was the same as that of type 1. The sound thus produced was named type 2. Next, the polarity of each type 1 sample was randomly flipped to disrupt the frequency structure of type 1, and the sound was filtered through a 20–20,000 Hz band pass filter in accordance with human aural characteristics. The resulting sound was named type 3. These sounds can sufficiently induce a finger BVPR (Ooishi and Kashino, 2012). The use of different sound stimuli is important because sound-induced vasoconstriction would become habituated when the same sound stimulus is listened to repeatedly (Maltzman et al., 1979). The sound pressure level (SPL) was modulated to 70 dB by measuring the maximum A-weighted SPL of these sounds in the slow mode. Each of these three types of sounds lasted for 1 min. The stimuli were converted to analog signals with an audio interface (EDIROL UA-5, Roland, Japan) and presented through headphones (Sennheiser HD650, Germany).

### Physiological measurements

Finger photoplethysmographs (PPGs) were used to measure the BVP amplitude in the finger. Analog data were amplified and digitized with a BIOPAC MP150 (BIOPAC Systems, USA). The sampling rate was 1,250 Hz for the finger PPGs. Amplified analog data were processed with a high pass (0.5 Hz) filter. The PPG transducer was attached to the left index finger of each participant. The finger BVPR indicates the vasoconstriction of the arterioles at the finger mediated by α-adrenergic receptors (Grote et al., 2003).

### Endocrinological measurements

To measure the salivary levels of testosterone and cortisol, saliva samples (2 mL/person) were directly collected in conical tubes from the participants between 15:00 and 16:00 h. All the samples were immediately stored at −80°C until needed for the measurement. The testosterone and cortisol concentrations in the saliva were determined using a liquid chromatography-tandem mass spectrometry (LC-MS/MS) system. All the analyses of the testosterone and cortisol levels in saliva were performed using the standard protocols by ASKA Pharma Medical Co. Ltd. (Japan) (Matsui et al., 2009; Yamashita et al., 2009), which has significant experience as regards various types of steroid hormonal assay. Staff at the company were not informed of the sample content or the nature of the study.

### Experimental procedure

The participants were given general information about the experiment, and their written consent was obtained in advance. They were instructed not to drink anything including alcohol or caffeine from 20:00 h on the day before their participation, and not to eat or drink anything other than still water after lunch (12:00–13:00) on the day of the experiment. They sat on a sofa wearing headphones, and were attached to PPG transducer electrodes for 10 min to adapt them to the experimental environment. Before the listening session, 2 mL saliva samples were collected from the participants via passive drool into a cold tube for hormone measurements. When the listening session started, the participants were presented with one of the experimental sounds, types 1, 2, and 3, for 1 min. The participants came to the laboratory for 3 days, and on each day they listened to one of the sound types and a saliva sample was collected once before the listening session. In summary, the participants listened to the sound and provided a saliva sample three times. We arranged for each participant to come to the laboratory at the same time on each of the 3 days. The sound type order was randomized.

### Data analysis and statistical evaluation

**Figure 1:**
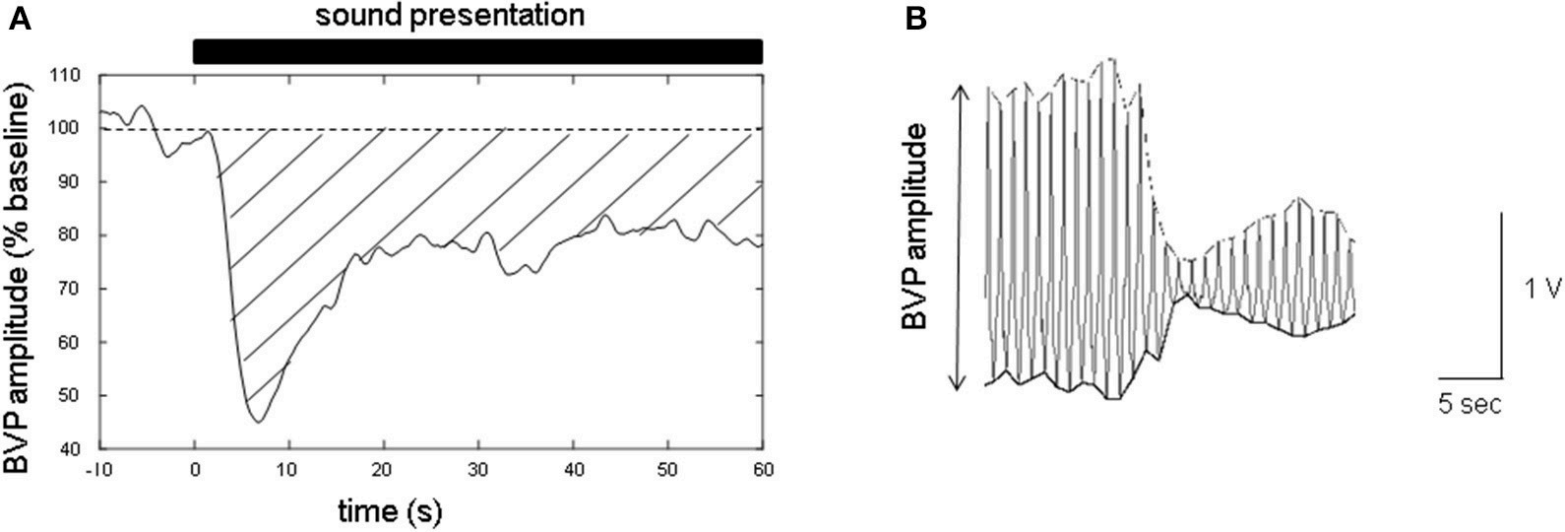
Sample of the data of the blood volume pulse (BVP) amplitude measured with photoplethysmographs (PPGs) at fingertip. **(A)** The time series of the finger BVP amplitude. The baseline was set at 100%. The closed bar above the graph indicates the sound presentation period. The time at the onset of the sound stimuli is defined as *t* = 0 s. The area between the dashed line (baseline, 100%) and the solid line (BVP amplitude curve) covered with a transverse line indicates the degree of the sound-induced finger BVP response (finger BVPR). If the BVP amplitude curve is below the baseline, the sign of the area is defined as plus. **(B)** Sample recording of the waveform of the finger PPGs. To evaluate the finger BVP amplitude, both the positive and negative peaks of the finger PPG waveform were resampled at 100 Hz by linear spline interpolation.

The time-series data of the finger BVP amplitude were expressed as a percentage of the mean value during baseline recording (for 10 s before the presentation of the sound stimuli; Figure 1A). To measure the BVP amplitude, the time series of the positive and negative peak values for each PPG measurement were resampled at 100 Hz by linear spline interpolation (Figure 1B). The finger BVPR was determined by calculating the mean reduction between *t* = 3 and *t* = 60 s for each experiment, in which the area under the baseline (100%) and over the curve of normalized BVP amplitude was calculated and this value was divided by 57 s (Figure 1A). The difference between the finger BVPRs for different sound types was analyzed with one-factor repeated measures ANOVA with sound type (types 1–3) as a within-subject factor.

To evaluate the relationship between the testosterone/cortisol ratio and the finger BVPR, within-subject and between-subject analysis were performed. In the within-subject analysis, we performed two analyses. First, Pearson's correlation coefficient R between the cortisol level, testosterone level, or the testosterone/cortisol ratio and the finger BVPR was calculated for three sound stimuli within each participant, and we evaluated the significant difference between the calculated R and no correlation (*R* = 0) with a paired *t*-test. Second, the finger BVPR was labeled “low,” “mid,” or “high” according to the cortisol level, testosterone level, or testosterone/cortisol ratio, respectively. The significant difference in the finger BVPR between low, mid, and high was evaluated with one-way repeated-measures ANOVA. In the between-subject analysis, the finger BVPRs, cortisol levels, testosterone levels, and testosterone/cortisol ratios for three sound stimuli were averaged for each participant. The statistical correlation between the average cortisol level, testosterone level, or testosterone/cortisol ratio and the average finger BVPR was examined with Pearson's correlation method.

Multiple comparisons between three levels of cortisol, testosterone, and testosterone/cortisol ratio (low, mid, and high) were analyzed with Ryan's method.

The significance was defined as *p* < 0.05. Huynh–Feldt corrections were applied where appropriate.

## Results

### Difference between finger BVPRs of 3 sound stimuli

**Figure 2:**
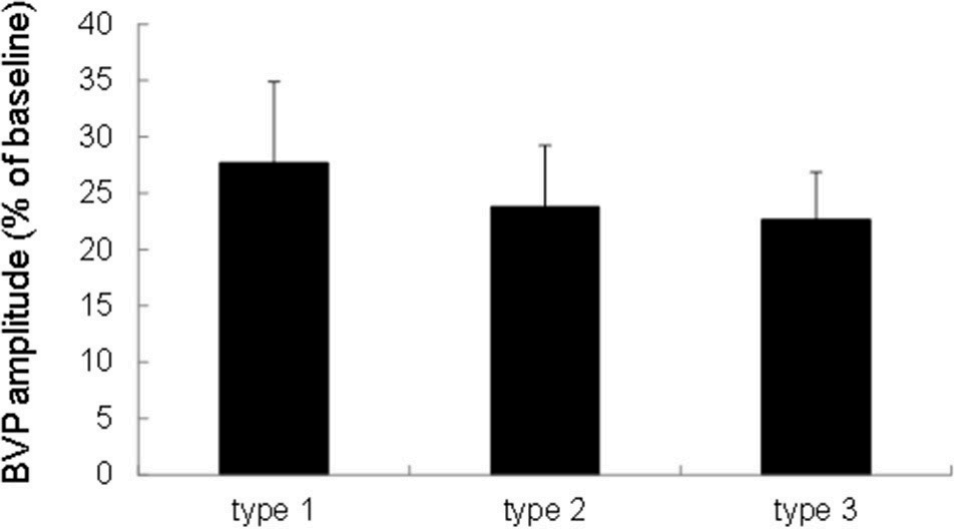
Comparison of the finger BVPR between three types of sounds. The data were represented as means ± S.E.M.

We performed a one-factor repeated measures ANOVA with Sound type as a within-subject factor. An ANOVA revealed that the finger BVPR for a sound stimulus was not significantly different for the three sound types [*F*_(2, 38)_ = 0.251, *p* = 0.779, Figure 2], which is consistent with the findings of a previous study (Ooishi and Kashino, 2012). This indicates that there is no sound-type-dependent alteration of the strength of vasoconstriction at the fingertip.

### Correlation of sound-induced finger BVPR with salivary cortisol level, testosterone level, and testosterone/cortisol ratio within participants

**Figure 3:**
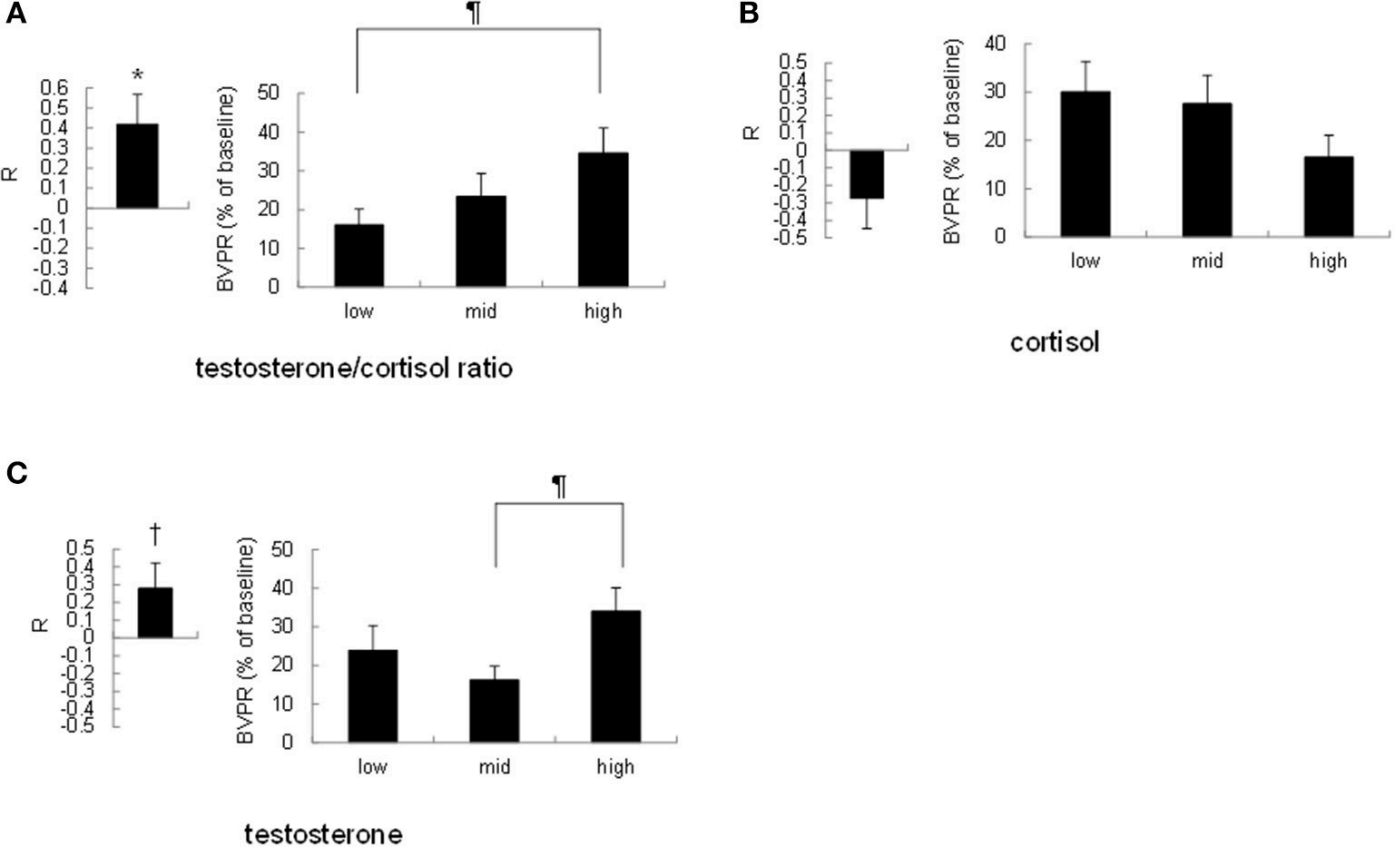
Correlation between the resting salivary hormone levels and the sound-induced finger BVPR within participants. **(A)** Left: the correlation coefficient R between the salivary testosterone/cortisol ratio and the sound-induced BVPR within each participant. Right: comparison of the sound-induced finger BVPR between low, mid, and high levels of testosterone/cortisol ratio. **(B)** Left: the correlation coefficient R between the salivary cortisol level and the sound-induced BVPR within each participant. Right: comparison of the sound-induced finger BVPR between low, mid, and high levels of cortisol. **(C)** Left: the correlation coefficient R between the salivary testosterone level and the sound-induced BVPR within each participant. Right: comparison of the sound-induced finger BVPR between low, mid, and high levels of testosterone. *p < 0.05, ^†^*p* < 0.1 for paired *t*-test; ^¶^*p* < 0.05 for Ryan's method of adjusting the *P*-value.

To investigate the possibility that the strength of the sound-induced vasoconstriction is correlated with the basal testosterone/cortisol ratio within participants, we performed the following two analyses. First, we calculated Pearson's correlation coefficient R between the cortisol level, testosterone level, or testosterone/cortisol ratio and the finger BVPR for three sound stimuli for each participant. Pearson's correlation coefficient R between the testosterone/cortisol ratio and the finger BVPR was significantly larger than no correlation (*R* = 0) [*t*_(19)_ = 2.84, *p* = 0.011, Figure 3A left], however the *R*-value between the salivary cortisol level and the finger BVPR was not significantly different from no correlation (*R* = 0) [*r*_(19)_ = 1.61, *p* = 0.12, Figure 3B left]. There was a trend toward a significance between no correlation (*R* = 0) and the *R*-value between the salivary testosterone and the finger BVPR [*t*_(19)_ = 1.93, *p* = 0.069, Figure 3C left]. Second, the finger BVPR was labeled “low,” “mid,” or “high” according to the size of the cortisol level, testosterone level, or testosterone/cortisol ratio, respectively. A one-way repeated-measures ANOVA revealed that a significant difference between low, mid, and high was observed for the testosterone/cortisol ratio [*F*_(2, 38)_ = 3.67, *p* = 0.035, partial η^2^ = 0.162, Figure 3A right] and testosterone [*F*_(2, 38)_ = 3.30, *p* = 0.048, partial η^2^ = 0.148 Figure 3C right], while no significant difference was observed for cortisol [*F*_(2, 38)_ = 2.040, *p* = 0.14, partial η^2^ = 0.097 Figure 3B right]. Ryan's method of adjusting the *P*-value showed that the finger BVPR in the high condition was larger than that in the low condition for the testosterone/cortisol ratio [*t*_(38)_ = 2.69, *p* = 0.032, Figure 3A right], and that the finger BVPR in the high condition was larger than that in the mid condition for testosterone [*t*_(38)_ = 2.56, *p* = 0.044, Figure 3C right].

Taken together, although the salivary testosterone level has some effect, these results suggest that the testosterone/cortisol ratio is the most effective factor as regards the sound-induced finger BVPR within participants.

### Correlation of sound-induced finger BVPR with salivary cortisol level, testosterone level, and testosterone/cortisol ratio between participants

**Figure 4:**
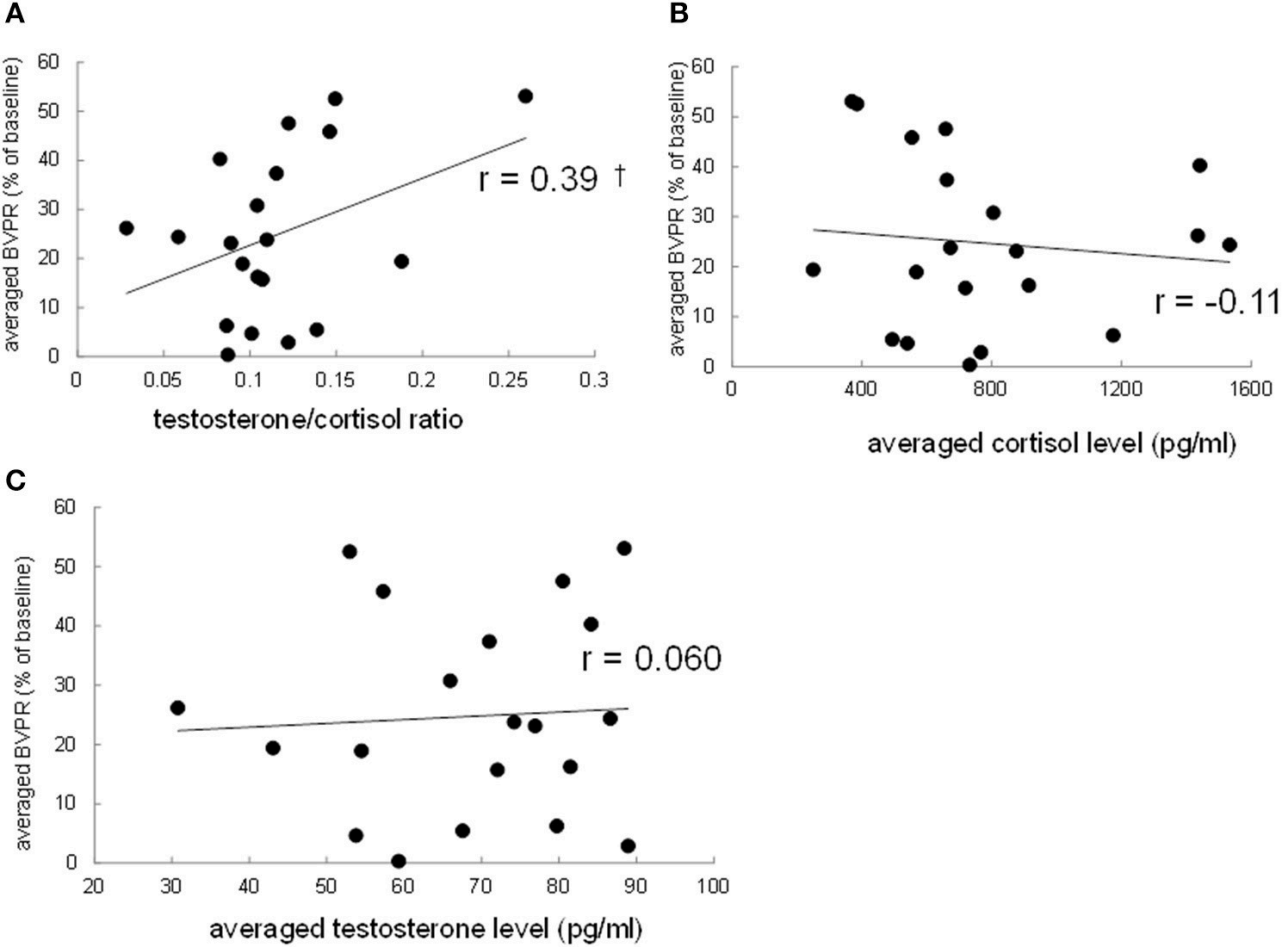
Correlation between the resting salivary hormone levels and the sound-induced finger BVPR between participants. **(A)** The salivary testosterone/cortisol ratio vs. the sound-induced BVPR, **(B)** the salivary cortisol vs. the sound-induced BVPR, and **(C)** the salivary testosterone level vs. the sound-induced BVPR. Pearson's correlation coefficient *r* is described. ^†^*p* < 0.1.

To investigate the possibility that the strength of the sound-induced vasoconstriction is correlated with the basal testosterone/cortisol ratio between participants, the finger BVPR, the salivary cortisol level, the salivary testosterone level, and testosterone/cortisol ratio were averaged, respectively, among three data for three sound stimuli for each participant. Pearson's correlation method revealed that there was a trend toward a significance of correlation between the average testosterone/cortisol ratio and the average finger BVPR (*r* = 0.39, *p* = 0.088, Figure 4A), while no significant correlation was observed between the average cortisol and the finger BVPR (*r* = −0.10, *p* = 0.65, Figure 4B) or between the average testosterone and the finger BVPR (*r* = 0.060, *p* = 0.80, Figure 4C). These results suggest that the testosterone/cortisol ratio has some relationship with the sound-induced BVPR between participants.

## Discussion

This study showed that sound-induced finger BVPR represented as vasoconstriction varies according to the resting testosterone/cortisol ratio. This is the first report demonstrating the relationship between the testosterone/cortisol ratio and the strength of sound-induced vasoconstriction. Interestingly, the correlation between sound-induced vasoconstriction and the salivary cortisol level, testosterone level, or testosterone/cortisol ratio is different for within-subject analysis and between-subject analysis. In within-subject analysis, the finger BVPR at a high testosterone/cortisol ratio was significantly larger than that at a low level (*p* = 0.032), the finger BVPR at a high testosterone level was significantly larger than that at a mid-level (*p* = 0.044), however there was no significant difference between the low and high levels in terms of cortisol. Furthermore, the correlation coefficient between the finger BVPR and testosterone/cortisol ratio within participants was significantly larger than no correlation (*p* = 0.011), while the trend of the difference between the correlation coefficient between the finger BVPR and testosterone within participants and no correlation remained toward significance (*p* = 0.069). In between-subject analysis, there was a trend toward significance for the correlation between the finger BVPR and the testosterone/cortisol ratio (*r* = 0.39, *p* = 0.088), while no correlation was observed between the finger BVPR and testosterone (*r* = 0.060, *p* = 0.80). These results suggest that although sound-induced vasoconstriction is affected by testosterone within participants, the testosterone/cortisol ratio has the largest correlation with the sound-induced vasoconstriction both within participants and between participants.

To evaluate the strength of the activation of noradrenergic sympathetic nerves induced by sound stimuli, we measured the vasoconstriction at a fingertip with photoplethysmography. Earlier studies have examined the autonomic responses by monitoring the change in the HR (Rimmele et al., 2007) and the heart rate variability (HRV) (Iwanaga et al., 2005). However, it is difficult to extract the contribution of the noradrenergic sympathetic nerves from these measurements. HR is controlled by at least three types of premotor neurons located in the RVLM, the nucleus ambiguus (NA), and the dorsal motor nucleus of vagus (DMNX), respectively. Moreover, the accelerating effects of RVLM, NA, and DMNX have a decelerating effect on the HR. The vagal control of the HR by NA and DMNX is called polyvagal (Porges, 1995). Regardless of the complexity of the control of the HR, the sound stimuli used in this study induce HR reduction only (Ooishi and Kashino, 2012), which means that it is impossible to extract the effect of the sympathetic control of the HR. The application of HRV analysis has been thought to make it possible to evaluate the strength of the vagal cardiac control by NA (Porges, 1995) and the simpathovagal balance (1996). A previous review indicated that at least 2 min of continuous HR data series are needed to evaluate the sympathovagal balance, which means that HRV analysis is not suitable for the transient activation of the sympathetic nerves such as the current study (1996). In contrast, the measurement of vasoconstriction at a fingertip is useful for the following reasons. First, the vasoconstriction induced by sound stimuli can be monitored second-by-second (Figure 1). Second, the vasoconstriction of the arterioles at the fingertip is derived from pure noradrenergic activity mediated by α-adrenergic receptors (Grote et al., 2003). Therefore, we can evaluate the strength of pure noradrenergic sympathetic nerve activity by measuring the decrease in finger BVP amplitude without any modulation by parasympathetic nerve activity. Many previous studies have measured the vasoconstriction at a fingertip as an index of the activation of the sympathetic nerves (Awad et al., 2001; Grote et al., 2003; Iani et al., 2004; Salimpoor et al., 2009, 2011; Ooishi and Kashino, 2012; Sato and Ooishi, 2012).

When considering the neural mechanism of sound-induced vasoconstriction, it is important to note that the RVLM and the amygdala might be involved in such mechanism. The RVLM receives a direct projection (Saha et al., 2005) and an indirect projection through the nucleus tractus solitaries from the CeA (Saha, 2005), which contribute to the activation of neurons in the RVLM. On the other hand, the amygdala is also affected by the auditory system. A previous study suggests neural connections between the MGB in the thalamus and the LA because auditory stimulation induces an increase in the firing rate of neurons in the LA as well as in the MGB (Bordi and LeDoux, 1992). In addition, the AC has neuronal projections to the LA (Kraus and Canlon, 2012). Taken together, the amygdala-RVLM relationship might be a neural regulator that induces sound-induced vasoconstriction.

The testosterone/cortisol ratio has been regarded as a biomarker for human social aggression (Terburg et al., 2009). When the testosterone level is high and the cortisol level is low, humans are prone to respond to environmental stimuli. This can be explained with the motivational imbalance model (Arnett, 1997), in which the behavioral inhibition and behavioral activation systems interact. The behavioral inhibition system prevents a person from acting to avoid possible punishment, while the behavioral activation system drives a person to seek a possible reward. Earlier studies demonstrated that testosterone facilitates vasopression gene expression in the amygdala (Szot and Dorsa, 1994), and the vasopression receptor gene expression in the amygdala is positively correlated with the social interaction time (Murakami et al., 2011), from which it is suggested that the testosterone action on the amygdala facilitates the approaching behavior. On the other hand, cortisol increases the expression of the corticotropin releasing hormone gene in the amygdala, resulting in inhibited behaviors (Erickson et al., 2003). These findings suggest that testosterone and cortisol are the chemical bases of the behavioral activation and behavioral inhibition systems, respectively. Taken together, the testosterone/cortisol ratio is an index of responsivity to environmental stimuli through the amygdala activity. Considering the neural connection between the amygdala and the RVLM (Saha, 2005; Saha et al., 2005), the current results are consistent with this model in the sense that a higher testosterone/cortisol ratio can facilitate a larger finger BVPR to sound stimuli. In particular, the amygdala receives direct neural projections from the MGB (Bordi and LeDoux, 1992) and the AC (Kraus and Canlon, 2012), both of which are involved in the main ascending stream of auditory processing, meaning that the amygdala is possibly a region that relates the basal testosterone/cortisol ratio to the sound-induced finger BVPR.

## Conclusion

The current study revealed a significant correlation between sound-induced finger BVPR and the resting testosterone/cortisol ratio within participants, while also noting a trend toward the significance of this correlation between participants. Although testosterone affected the finger BVPR within participants, this effect disappeared between participants. These results suggest that the testosterone/cortisol ratio can explain the cardiovascular activation induced by the approaching sound stimuli more efficiently than testosterone alone and cortisol alone.

## Limitations

The current study was designed to examine non-invasively the effect of the resting testosterone/cortisol ratio on sound-induced vasoconstriction mediated by the noradrenergic sympathetic activity with human subjects. We adopted the measurement of vasoconstriction at a fingertip with photoplethysmography as the most effective and reliable way to achieve our goal. However, there was no direct evidence of the contribution of the noradrenergic sympathetic nerves to the sound-induced vasoconstriction because we could not directly measure the activity of neurons located within the RVLM. Moreover, we could not undertake the pharmacological treatment of applying adrenaline-receptor antagonists to the participants. In addition, to achieve non-invasiveness, we measured the cortisol and testosterone levels from saliva samples. Although we asked the participants to take a 10-min rest period during which they could completely relax while sitting on a sofa without any significant sensory stimuli to allow them to adapt to the experimental environment, it is possible that the levels of steroids, glucocorticoid in particular, fluctuate rapidly in the brain due to an internal or external cause. There is room to debate whether the salivary cortisol and testosterone levels in the resting state could explain the effect of the subsequent psychological or psychophysiological tasks. Further studies using animal models could measure the activity of neurons in the RVLM electrophysiologically to directly monitor sympathetic cardiovascular control, collect cerebro-spinal fluid with microdialysis in real time to directly monitor the precise fluctuation in the glucocorticoid level in the brain, and apply several adrenaline receptor antagonists to determine the pathway by which the sound-induced vasoconstriction occurs.

In this study, females were not selected as participants to avoid any effects of the menstrual cycle. Given that the purpose of the study was to examine the effect of testosterone, cortisol, and the testosterone/cortisol ratio on sound-induced vasoconstriction, we wanted to exclude the possible effects of estradiol and progesterone. A further study could examine the effect of estradiol and progesterone on sound-induced vasoconstriction and its interaction with the effect of testosterone and cortisol by recruiting female participants and by examining the phase of their menstrual cycle on the day of the experiment.

## Author contributions

YO: designed this study, acquired and analyzed the data, and wrote the manuscript.

### Conflict of interest statement

The author declares that the research was conducted in the absence of any commercial or financial relationships that could be construed as a potential conflict of interest.
